# Protective potential of BM-MSC extracted Exosomes in a rat model of Alzheimer’s disease

**DOI:** 10.1371/journal.pone.0320883

**Published:** 2025-05-06

**Authors:** Atefeh Sadeghi, Maryam Noorbakhshnia, Shabanali Khodashenas

**Affiliations:** 1 Department of Plant and Animal Biology, Faculty of Biological Science and Technology, University of Isfahan, Isfahan, Iran; 2 Department of Medical Biotechnology, School of Advanced Technologies in Medicine, Thalassemia Research Center, Hemoglobinopathy Institute, Mazandaran University of Medical Sciences, Sari, Iran; University of Eastern Piedmont: Universita degli Studi del Piemonte Orientale Amedeo Avogadro, ITALY

## Abstract

Exosomes are extracellular vesicles, which are released into the extracellular space by all types of cells, especially stem cells. Compared with stem cells, exosomes are safer and can be considered one of the most promising therapeutic strategies for neurodegenerative disease. We examined the effect of exosomes derived from bone marrow mesenchymal stem cells (BM-MSC) on a rat model of Alzheimer’s disease (AD). For this purpose, male Wistar rats weighing 220–250 g were used. For the induction of AD, rats received a daily dosage of 100 mg/kg Aluminum chloride (Alcl3) by oral gavage for 60 days. Also, Primary BM-MSC was extracted from the femora of Wistar rats (male, 100–150 g). Extracted exosomes were Characterized and Qualified using TEM Microscope and Zetasizer Nano. Specific markers of exosomes were evaluated by Flow cytometry. MSC-extracted exosomes (150 µg/µl) were injected 2 or 5 times into the animals via tail vein on specific days. Our data revealed that receiving exosomes significantly prevented AlCl3-induced enhancement of hippocampal APP gene expression, beta-amyloid plaque formation, impairment of passive avoidance learning and spatial memory. However, exosome injections in healthy subjects caused some negative effects such as spatial memory impairment. It seems, MSC-derived exosomes can be considered as a candidate to prevent AD progression.

## Introduction

Alzheimer’s disease (AD) is the most common form of dementia in old age, although early-onset AD with less prevalent can happen much earlier. AD is a progressive neurological disease [1]. By 2021, about 50 million people around the world have been affected by dementia, which is expected to triple by 2050 due to the aging population. Of about 50 million people worldwide with dementia, 60% to 70% are estimated to have AD. The increase of this disease in the communities amplifies the burden of disease, health care, and costs [2]. Since AD is multifactorial, studies have addressed it in various aspects. For instance, it has been indicated that some traditional medicinal herbs via inducing autophagy can decrease Aβ pathology in AD models [3,4]. Deficit in lysosomal autophagy pathway has been proposed as a hallmark feature in AD [5]. However, despite all efforts, more research is essential. Furthermore, there is an urgent need to find new diagnostic agents with therapeutic properties that can render therapeutic functions. AD usually starts with short-term memory loss and worsens over time. As it progresses additional neuropsychiatric symptoms may manifest, including periods of confusion, disorientation, mood change, aggression, agitation, and eventually delusion/hallucination in later stages [6]. Patients with AD suffer from sever discomfort in life, decrease life expectancy, and low attention from society, which threatens them physically and mentally. AD is characterized by neuronal damage in the hippocampal tissue and cerebral cortex atrophy which can lead to brain weight loss [7–9]. The main cellular AD symptoms are intracellular neurofibrillary tangles (NFTs) and extracellular amyloid beta (Aβ) plaques [10]. Aβ peptides are generated through proteolytic cleavages of amyloid precursor protein (APP) by β- and γ-secretase enzymes. The majority forms of Aβ are Aβ40 and Aβ42, in which Aβ42 is more potent for deposition [11]. It has been indicated that enhancement of APP expression may enhance the risk of AD [12].

Aluminum (Al) is a potent neurotoxin that is involved in the onset and progression of several cognitive disorders such as AD. Pieces of evidence have shown that the accumulation of high amounts of Al in certain areas of the brain causes the production of toxic free radicals which can lead to neurodegenerative disorders [12,13]. It has been found that Al inhibits long-term potentiation, induces inflammatory responses, affects axonal transports, causes synaptic structural abnormalities, and intense memory loss [14]. Moreover, Aluminum chloride (AlCl3) injection is a common method in the laboratory to create an animal model of AD [15].

There is no definitive treatment for AD and available treatments only slow down the disease process [16] The most current pharmaceutical treatments for patients are memantine (an NMDA receptor antagonist) and acetylcholine esterase inhibitors including galantamine, donepezil, and rivastigmine [17,18]. In recent years, cell therapy and the use of stem cells as promising therapies have been considered [19]. Mesenchymal stem cells (MSCs) are one of the most accessible primary cells and can be easily harvested from a large variety of tissues, such as bone marrow. They have the potential to differentiate into different mesenchymal cells such as bone, fat, cartilage, muscle, and non-mesenchymal cells such as neurons, glial cells, etc. These properties have made the MSCs a suitable candidate for tissue engineering, nerve regeneration, and vascular tissue repair [20]. Shreds of evidence have shown that MSCs exert their therapeutic effects mainly through secretory membrane agents. In other words, the beneficial effects of MSCs on tissue repairing and regeneration are mediated by the paracrine activity of the secretory agents of these cells, which are named exosomes [21].

Exosomes are small membrane vesicles (40–100 nm diameter), secreted by various cells, and can almost be found in all biological body fluids such as blood, urine, breast milk, plasma, cerebrospinal fluid, and synovial fluid. Exosomes originate initially by invagination of the endosomal membrane to create multivesicular bodies (MVB), these vesicles are released to the extracellular environment after the fusion of late MVBs with the plasma membrane [19]. Exosome secretion from cells occurs in both physiological and pathological conditions. They contain different proteins, lipids, and nucleic acids including mRNA, microRNAs (miRNAs), and other non-coding RNAs (ncRNAs). Their composition is impressed by parent cells and can also be influenced by different cellular conditions or treatments [22]. Since, exosomes have beneficial effects on the parent cell, and nanoscale size, as well as their easy injection and management, they have opened a novel therapeutic perspective aimed at the development of cell-free strategies [21]. On the other hand, regarding that exosomes can cross the blood-brain barrier, their use for drug delivery in neurodegenerative diseases such as Alzheimer’s has gained significant attention in recent years [17].

Furthermore, the use of bone marrow mesenchymal stem cells (BM-MSC) derived exosomes for treating disease have several advantages over using these cells themselves. Exosomes do not proliferate in tissue and do not divide uncontrollably, unlike MSCs, whose proliferation and propagation in damaged tissue may lead to tumor formation. In addition, exosomes lack metabolism and have the least impact on their environment. The nanometer size of exosomes also reduces the possibility of their accumulation in blood vessels and the occurrence of blood clots. These vesicles can be sterilized and stored in the freezer for a long time without loss or reduction. In general, exosomes have the benefits of stem cells without their side effects [19]. The researchers highlighted the potential role of exosomes in complex synaptic and cell signaling and glial-neuronal interactions [23]. Masako et al. (2016) have shown that exosomes derived from mouse BM-MSC could improve cognitive impairment in diabetic mice by repairing damaged neurons and astrocytes [24]. Liu et al. (2021) have reported that BM-MSC extracted exosomes could decline cerebral ischemia injury and neuroinflammation and improve neurological function in a rat model of stroke [25].

Due to the lack of evidence about the effect of BM-MSC-derived exosomes against AD, this study aimed to evaluate the protective effect of these exosomes against Alcl3-induced neurotoxicity in an AD rat model by evaluating behavioral tests of learning and memory, hippocampal APP gene expression, and histological studies.

## Materials and methods

### Chemicals

Aluminum chloride was purchased from Merk Company.

### Exosome preparation

#### Isolation, culture, and characterization of MSCs.

Primary BM-MSC was extracted from the femora of Wistar rats (male, 100–150 g). Rats were gained from the Mazandaran University of Medical Science Laboratory Animal Research Center (Sari, Iran) and kept under specific pathogen-free conditions. Bone marrow was flushed under aseptic conditions using Dulbecco’s Modified Eagle Medium (Gibco, USA). The collected cells were washed 3 times with PBS, resuspended in DMEM containing 10% fetal bovine serum (Gibco, USA) and 1% L-glutamine (Gibco, USA), and seeded at 1 × 106 cells/ml into culture flasks. Cells were preserved in a humidified incubator (% 95) with 5% CO2 at 37 °C. Suspended cells were discarded after 48 h and then the medium changed every 3–4 days. When cells reached approximately 90% confluence they were harvested and diluted 1:2 or 1:3 at each passage. BM-MSCs used in all in vivo studies were between passages 3 and 10.

When MSCs reached the third generation, were characterized by Flow cytometry [26]. Briefly, cells were detached from the cell culture plate using trypsin/EDTA and shared into several tubes each containing about 4 × 105 single cells. Then centrifuge (300 g for 5 min) was performed and pellets were rinsed in 1 ml human serum. Cells kept 30 min at 4 ºC and then centrifuged at 1200 g for 10 min and resuspended in goat serum (3% (v/v) and incubated with CD105, CD90, CD73, and CD34 fluorochrome-labeled antibodies, as well as fluorescent isothiocyanate (FITC)-conjugated mouse anti-human CD45 (Biosciences, USA) for 60 min on ice. Thereafter, cells were centrifuged (1200 g for 5 min) and suspended in 200 μl of PBS. Finally, samples were evaluated using flow cytometry (Attune™ Acoustic Focusing Cytometer) and FlowJo® software.

#### Purification of exosomes.

Exosomes were purified from MSC cells and cultured in DMEM containing 10% exosome-free FBS. The conditional media were collected periodically at 48-hour intervals and exosomes were purified by conventional sequential centrifugation [27].

Briefly, MSC-conditioned medium was harvested at 48-hour intervals and was centrifuged (sigma, Germany) at 300 g for 10 minutes to eliminate suspended cells and debris. The supernatant was centrifuged at 2000 g for 10 min and then, the remaining supernatant centrifuged at 10,000 g for 30 min. Next, ultra-centrifuge (Backman, USA) was performed at 100,000 g for 70 min. After throwing away the supernatant, the pellet was washed once in PBS and then ultra-centrifuge (at 10,000 g for 30 min) was performed again. Finally, the exosomes pellet was suspended in PBS and aliquots of exosomes were stored at -80 ºC. In the next step quantification of exosomes was performed by flow cytometry and specific antibodies for CD9, CD63, and CD81 according to the manufacturer’s instructions (Baft Azma, Iran).

#### Preparation and characterization of exosomes by electron microscopy.

Ultra-concentrated BM-MSC exosomes were fixed in 4% paraformaldehyde in PBS. The samples were air dried on Formvar-carbon coated EM grids and stained with 0.5% uranyl acetate in 30% ethanol for 10 min and then stained with lead citrate for 10 min. Removing of Lead precipitates on grid sections was performed by rinsing in NaOH (0.05 M) and then rinsing in distilled H2O. For drying, grids were left at room temperature and finally, the stained sections were observed under a transmission electron microscope (Philips, Nederland) operated at 150 kV.

#### Determination of exosome size.

The size of the isolated exosomes was measured using a Dynamic light scattering (DLS) Nanoparticle Analyzer, nanoPartica SZ-100, according to the manufacturer’s instructions (HORIBA Co. England).

### Animals

Thirty-six Wistar rats (220–250 g) were randomly selected from the population of intact rat nests at the University of Isfahan. Rats were housed in six cages in a temperature (23 ◦C) controlled environment. The animals were kept in standard condition with a 12 h light/12 h dark cycle (light on at 07:00 a.m.). Rats had free access to water and food in their home cage. The ethical aspects of the project were approved by the graduate studies committee of the Department of Plant and Animal Biology, University of Isfahan according to the NIH Guide for the Care and Use of Laboratory Animals and following ARRIVE guidelines (ethical approval ID: IR-UI-REC.1403.113). Training and testing were performed between 8:00 and 12:00 h. Cage beds were covered with shredded wood, which was cleaned and refreshed every 3 days. Then, rats were divided into six groups randomly, with six rats per group.

**Fig 1:**
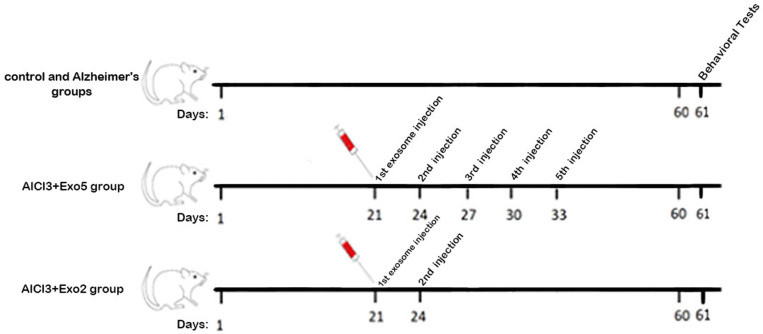
Timeline of experiments.

To control the similarity of administrations among 6 groups, all groups received 60 days of AlCl3 or saline by oral gavage and 2 or 5 times of exosome (150µg/µl) or saline by intra-tail vein injection. The dosage and protocol of exosome injection were performed according to previous studies [28] and also our pilot studies. The timeline of experiments is shown in Fig 1.

Groups were categorized as follows:

Group 1: Saline (control) group received saline (1ml/kg) for 60 continuous days by oral gavage.

Group 2: The Alcl3 group received AlCl3 (100 mg/kg/day) over 60 days through gavage.

Group 3: Alcl3 + exosome [2] group received AlCl3 (100 mg/kg/day) over 60 days and exosome, 2 times on days 21 and 24, by injection into the tail vein.

Group 4: Alcl3 + exosome [5] group received AlCl3 (100 mg/kg/day) for 60 continuous days and exosome, 5 times on days 21–33, once every three days by administration into the tail vein.

Group 5: Exosome [2] group received saline via gavage for 60 days and exosome, 2 times on days 21 and 24, by injection into the tail vein.

Group 6: Exosome [5] group received saline by oral gavage for 60 days and exosome, 5 times on days 21–33, once every three days by injection into the tail vein.

### Behavioral tests

For evaluating learning and memory two behavioral tests including Morris water maze and shuttle box were used. Behavioral tests started the day after the last treatment.

### Morris water maze

This device was created in 1982 by a scientist named Morris. Using this maze, rats are trained to find a specific place in space only with out-of-device visual guides. The device used in this study consisted of a black cylindrical pool with a diameter of 150 cm and a depth of 60 cm, which was filled to a height of 40 cm with water at a temperature of 25 ± 2ºC. The pool was hypothetically divided into four quadrilaterals including NW (northwest) as quadrant no. 1, SW (southwest) as quadrant no. 2, SE (southeast) as quadrant no. 3, and NE (northeast) as quadrant no. 4. A Plexiglas platform with a diameter of 12 cm was placed in the center of the quarter circle southwest of the pool. The platform was about 1 cm below the water surface and the platform was hidden from the view of animals. The position of the platform remained the same on all training days. Attached to the walls of the experimental room were three visual guides in the form of circles, squares, and triangles, which the animal could use to find the position of the hidden platform. The animal’s movements and behavior were tracked and monitored by an infrared camera located two meters above the center of the pool. Moreover, using a computerized tracking system all movements of rats with details (Video Tracking Software, designed by BorjSanat Company, Iran) were tracked.

This test was performed in three stages over three continuous days. On the first day, to make the animal accustomed to the environment and conditions, the rat was released to the pool once and returned to the cage after a minute of swimming. Next-day training was performed and a single training protocol with 8 trials was used [29]. The single training session consisted of eight trials with four different starting positions that were equally distributed around the perimeter of the maze. In this phase, the animal was randomly released from all four areas of the pool into the water twice and was allowed to swim for one minute to find the hidden platform and sit on it. After finding the platform, the animal was allowed to stay on it for 30 seconds. If the animal was unable to find the platform at the appointed time, it was led to the platform by hand. After each training, the animal was taken out of the pool, dried with a towel, and returned to the cage. On the third day, the probe test was performed. The platform was removed and the animal was once released from quarter 4, in front of the previous location of the platform, into the water and was allowed to swim for 60 seconds. Time spent in the target quadrant in which the platform was placed on a training day was recorded. The amount of time that animals spend in the target quadrant shows the level of memory retrieval [30].

### Passive avoidance learning test

A shuttle box was used for evaluating passive avoidance learning and memory. This device consisted of two compartments of the same size with a length and width of 20 and a height of 30 cm. One of the compartments was light and the other was dark, and they were connected by a guillotine door. The bottom of both chambers was made of stainless-steel rods. The rods in the dark chamber were connected to the electroshock system. The passive avoidance test strategy is based on the desire of rats to stay in a dark place. This test was performed in two days. On the first day (acquisition trial), to accustom the animal to the new conditions, rat was placed in the lighted compartment, facing away from the guillotine door. After 5 seconds, the door was opened, and the animal was allowed to enter the dark compartment. After 30 seconds, the animal was taken out and returned to its cage (this was performed for the habituation of the animal to the shuttle box). 30 minutes later the learning stage was performed. In this stage, the animal was placed in a lighted compartment. After 5 seconds, the door was opened and the delay to enter the dark compartment was recorded for each rat. Immediately after entering the dark compartment, the door was closed, and the rat received an electric shock (1.1 mA at a frequency of 50 Hz for 1 second) through the floor bars. After receiving the shock, rat was allowed to stay in the dark compartment for 30 seconds, then the animal was transferred to its cage. Two minutes later, the procedure was repeated until the animal did not move to the desired (dark) compartment for 12 consecutive seconds. On the second day, the animal was placed in the light compartment as on the acquisition day. After 15 seconds the door was opened and the delay time in entering the dark compartment was recorded (step-through latency, STL). Also, the elapsed time in each chamber was checked for 600 seconds and the time spent in the dark compartment, TDC was recorded [31–33].

Following behavioral tests, to check the ratio of brain weight to body weight, each rat was weighed, then anesthetized and sacrificed using a rodent guillotine. Immediately, the brains of animals were completely removed and weighed. The hippocampi of five animals from each group were separated on ice and transferred to liquid nitrogen. Then the hippocampi were kept at -70 ºC for molecular studies. Also, in each group, one brain was fixed in a 10% formalin solution for histological studies.

### Molecular study (Real-Time PCR)

#### mRNA quantitative assessment and cDNA synthesis.

RNA of each hippocampal sample was extracted using an RNX-plus kit (Cinnagen, Tehran, Iran) according to the manufacturer’s instructions. First, 250 μl of sterile PBS was added to the 100 mg hippocampal samples and the tissue was homogenized. 500 μl of RNX-plus was added to 50 μl of the mixture, and the mixture was vortexed for 15 seconds and then incubated on ice for 5 minutes. Then 200 μl of chloroform was added and the mixture was shaken for 15 seconds. It was placed on ice for 15 seconds. The mixture was then centrifuged at 12,000 rpm for 15 minutes at 4 °C. From the final mixture, 200 μl of the supernatant was transferred to sterile RNase-free microtubes and then 200 μl of isopropanol was added. After shaking gently, the mixture was incubated on ice for 15 minutes and centrifuged at 12,000 rpm for 15 minutes at 4 °C. After centrifugation and observation of sediment, the supernatant solution was discarded and the precipitate was washed with 1000 μl of 75% ethanol, then centrifuged at 7500 rpm at 4 °C for 4 minutes. The ethanol was then completely discarded and the microtube was dried at room temperature. Then 25 μl of DEPC-treated water was added and placed in BenMarie at 55–60 °C for 10 minutes. The extracted RNA concentration was determined using a Nanodrop spectrophotometer (Nanodrop Spectrophotometer manufactured by Thermo Scientific) and its purity was determined by examining the absorption ratio of 260/280 and the ratio of 260/230. Agarose gel electrophoresis was also used to evaluate the quality of the extracted RNA. The results of these two tests confirmed the quality and accuracy of the extracted RNA. DNase I treatment was applied using an RNase**-**free DNase I kit from Cinna Gen company. RNA was then converted to cDNA using the Cinna Gen cDNA synthesis kit and stored at – 20 ºC.

Primers were designed using Beacon Designer 7.5 and oligo6 software to amplify 134-bp amplification for APP, and 134-bp for β-actin an internal reference gene (Table 1). Quantitative real-time PCR (Real-Time PCR) was performed using RealQ Plus Master Mix Green kit (from Amplicon Company) and according to the protocol, the samples were diluted with DEPC water in a ratio of 1–100 and 2 μl of the sample with 5 μl of master mix and 1 μl of each primer were added to specific strips, and after spin placed in the Real**-**time PCR machine (ABI, USA). The expression of the APP gene as one of the most genes related to AD was examined and the β-actin gene was used as a housekeeping gene to normalize the APP gene expression. Finally, the results were analyzed by comparative method $2^{-\Delta \Delta Ct}$ and the melting curve of the samples was analyzed [32,34].

**Table 1:** Primer sequences for APP and β-actin.

Gen Forward Primer (5’- 3’) Reverse Primer (5’3’) Product length (bp)
APP TACTGCCAAGAGGTCTAC CGGTAAGGAATCACGATG 134 β-actin CTCTATGCCAACACAGTG AGGAGGAGCAATGATCTT 123

### Tissue processing

For staining Beta-amyloid plaques Bielschowsky’s Silver Staining was used. Furthermore, deparaffinized slides were placed in silver nitrate solution (Sigma-209139) at 10% (40°C) and stained for 15 minutes. Then the slides were washed 3 times with distilled water. Ammonium hydroxide (6-21-1336-Sigma) was added until the sediment formed completely transparent. Then the slides were re-stained in an ammonium silver solution in a 40°C oven for 30 minutes. Slides were placed in the working developer solution for about 1 minute or less. To prevent the reaction of silver, the probes were immersed in 1% ammonium hydroxide solution for 1 minute and the probes were washed with distilled water. Then, the samples were placed in sodium thiosulfate solution (Sigma-217263) (5%) for 5 minutes, and were washed three times with distilled water in the last step, the samples were dehydrated with alcohol and passed through Xylol [35,36].

### Statistical analysis

All data were presented as Mean ± SEM. Because of normal distribution and variance homogeneity, the results were analyzed using one-way ANOVA. Tukey’s post-test was used for comparison between experimental groups. In all cases, p≤0.05 was considered a significance level.

## Results

### Characterization of undifferentiated BM-MSCs

**Fig 2:**
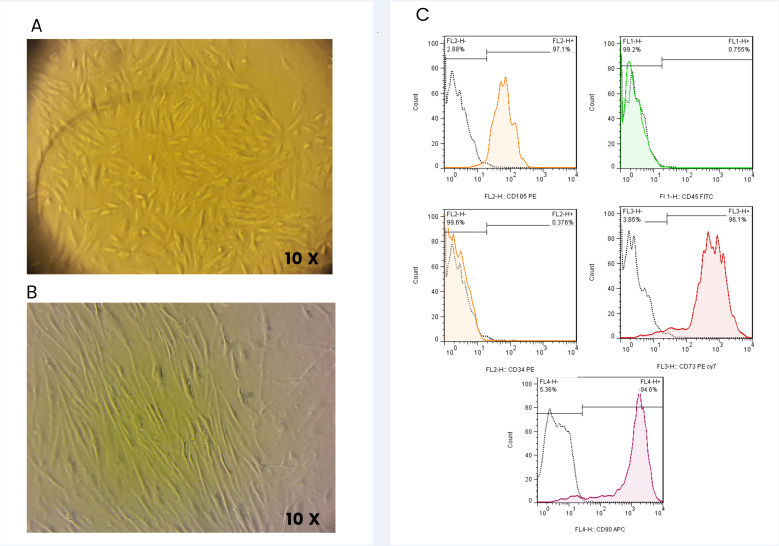
Isolation and Identification of BM-MSCs. 2A: light microscopic image of BM-MSCs 10 days after isolation from bone marrow. 2B: light microscopic image of BM-MSC in third passage after isolation, MSc cells morphologically had spindle-like shape. 2C: Flow cytometric analysis revealed that MSCs were uniformly negative for the hematopoietic markers including, CD34 and CD45, and positive for typical MSC antigens such as CD73 (96%), CD90(94%) and CD105(97%).

Light microscopic was used for the characterization of undifferentiated BM-MSCs. In passage 3, BM-MSCs were in a spindle-like shape under an inverted microscope. Also, flow cytometric analysis showed that specific cell surface markers including CD73 (99.7%), CD90 (88.8%), and CD105 (99.8%) were highly expressed while the expression of CD45 (1.6%) and CD34 (1.6%) was very low (Fig 2).

### Qualification of exosomes

**Fig 3:**
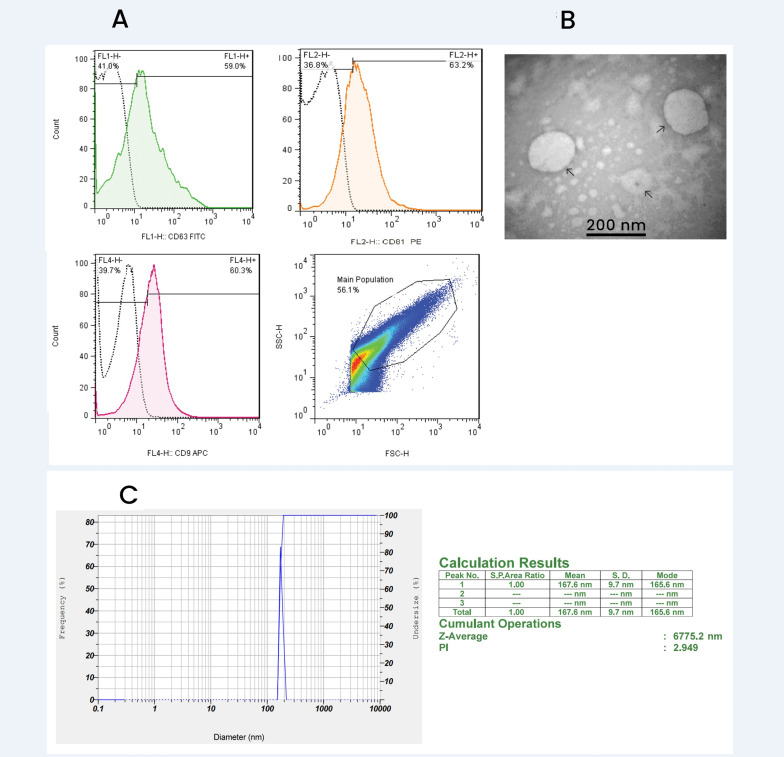
Characterization of isolated exosomes. 3A: flow cytometric analysis showed that All isolated exosomes expressed 3 special exosome markers including, CD9(63%), CD63 (60%), and CD81(63%). 3B: TEM imaging of Isolated exosomes showed a cup-shaped morphology and exosome size. 3C: sharp particle size and distribution peak at 167 nm of isolated exosomes was shown using DLS analysis.

MSC-extract exosomes were qualified using three methods including Transmission electron microscopy (TEM), zeta sizer, and flow cytometry. TEM was used for detecting the morphology of exosomes. They had caps-like shapes and more than 90% of them had 50–200 nm diameters in the TEM micrograph (Fig 3B). Dynamic light scattering (DLS) was utilized to estimate exosome size distribution and diameter. The isolated nano-vesicles had a size of 50–200 nm and a sharp peak at 109 nm. Moreover, DLS results were consistent with the size of the exosomes (Fig 3C). Finally, flow cytometric analysis was performed and expression of exosomal specific proteins, CD9, CD63, and CD81 was evaluated. Flow cytometry results showed 59% of the expression of CD63, 63.2% of CD81, and 60% of CD9 (fig 3A).

### Effect of treatments on brain weight

**Fig 4:**
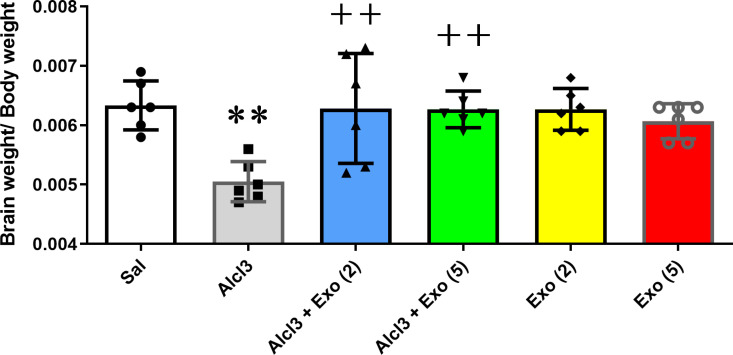
Rate of brain weight to body weight in different experimental groups. AlCl3 treatment caused a significant decrease in brain-to-body weight with respect to the control group (P < 0.01). 2 or 5 injections of BM-MSC extracted exosomes significantly protected against the deficit effect of Alcl3 on brain-to-body weight rate. Each value represents mean ± SEM, n = 6, **p < 0.01 vs. saline group and ^++^p < 0.01 vs. Alcl3 group. *Abbreviation*s: Sal, Saline; Exo [2], Two injections of exosomes; Exo [5], five injections of exosomes.

One-way ANOVA showed a significant difference in brain weight relative to body weight among experimental groups (F (5,30) =6.12, p = 0.0005). As expected, the Alzheimer’s disease (AlCl3) group showed a significant decrease in brain-to-body weight compared with the control group (p < 0.01). On the other hand, 2- or 5 injections of exosomes (150µg/µl) significantly prevented AlCl3-induced changes in brain weight (p < 0.01). Moreover, 2- or 5 injections of exosomes in healthy rats did not have a significant effect on brain weight to saline-treated rats (Fig 4).

### Effect of treatments on behavioral performance

#### Morris water maze.

**Fig 5:**
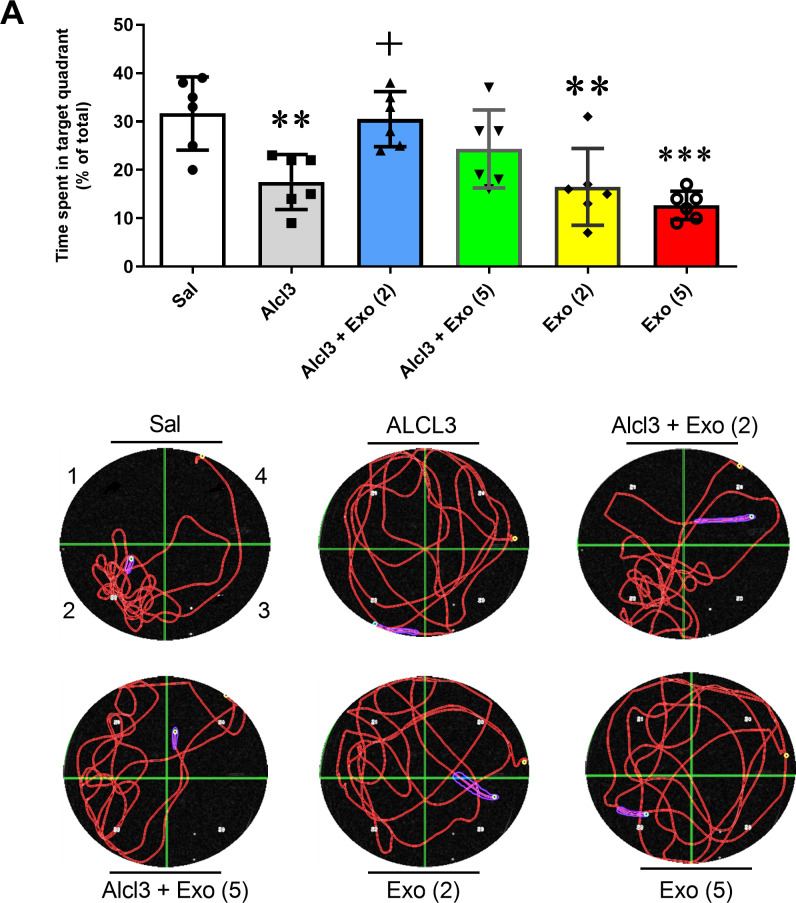
Spatial memory assessment in Morris water maze test. 5A: Effect of different treatments on time spent in the target zone. Alcl3 significantly decreased the time spent in the target zone with respect to the saline-treated group (p < 0.01). Two injections of exosomes prevented the impairment effect of Alcl3 on spatial memory compared to the Alcl3-treated group (p < 0.05). Two or five injections of exosomes in healthy rats impaired spatial memory. 5B: The swimming trajectory of each group in the probe test. No.2 was the quadrant area. Each value represents mean ± SEM, n = 6, **p < 0.01 and ***p < .001 vs. saline group and ^+^p < 0.05 vs. Alcl3 group. *Abbreviation*s: Sal, Saline; Exo [2], Two injections of exosomes; Exo [5], Five injections of exosomes.

One-way ANOVA revealed a significant difference in the percentage of time spent in target quadrants between different experimental groups (F (5,30) = 8.58, p < 0.0001). Alcl3 treatment significantly reduced the residence time of rats in the target quarter with respect to the control group (p < 0.01). Two injections of exosomes, but not five, significantly prevented AlCl3-induced changes in the time spent in the target quarter (p < 0.05). This data suggests the effectiveness of exosomes with a specified number of injections for protecting AlCl3-induced spatial memory defects. Moreover, two or five injections of exosomes into healthy rats (saline-treated rats) significantly reduced the residence time in the target quadrant compared to the control (saline) group. Therefore, it appears that exosome therapy cannot help to enhance spatial memory in healthy conditions (Fig 5).

#### Passive avoidance test.

Step-through latency (STL) and the time spent in the dark compartment (TDC) were evaluated at the retrieval test which was performed 24 hours after training. In the passive avoidance learning and memory test, declines in the STL and/or enhancements in the TDC express/expresses destruction of memory.

**Fig 6:**
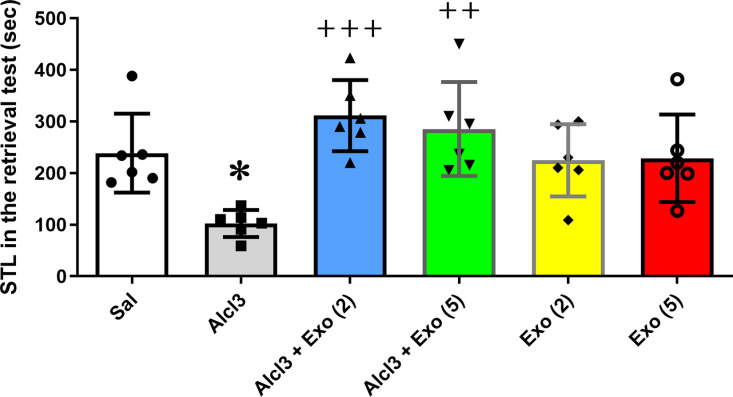
Effect of different treatments on step-through latency (STL) in the retention test of passive avoidance learning. Alcl3 significantly reduced STL with respect to the saline group (p < 0.05). Two or five injections of MSC-extracted exosomes significantly prevented the impairment effect of ALcl3 on STL. Two or five injections of exosomes to healthy animals did not show a significant effect on STL with respect to the saline group (P > 0.05). Each value represents mean ± SEM, n = 6, * p < 0.05 vs. saline group ^++^p < 0.01 and ^+++^p < .001 vs. Alc3 group. *Abbreviation*s: Sal, Saline; Exo [2], Two injections of exosomes; Exo [5], Five injections of exosomes.

**Fig 7:**
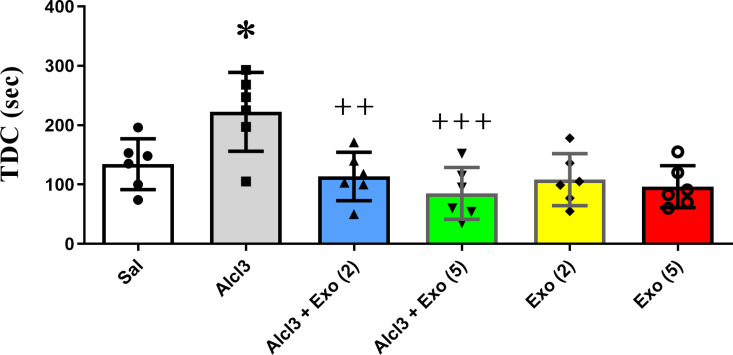
Effect of different treatments on time spent in the dark compartment (TDC) of passive avoidance learning. Alcl3 significantly enhanced TDC with respect to the saline group (p < 0.05). Two or five injections of MSC-extracted exosomes significantly prevented the impairment effect of ALcl3 on TDC. Two or five injections of exosomes to healthy animals did not have significant effect on TDC compared to the saline group (P > 0.05). Each value represents mean ± SEM, n = 6, * p < 0.05 vs. saline group ^++^p < 0.01 and ^+++^p < .001 vs. Alc3 group. *Abbreviation*s: Sal, Saline; Exo [2], Two injections of exosomes; Exo [5], Five injections of exosomes.

One-way ANOVA revealed a significant difference between six different experimental groups, STL (F (5,30) = 5.92, p = 0.006) and TDC (F (5,30) = 6.86, p < 0.001). Tukey post hoc analysis test showed that AlCl3 significantly reduced STL (P < 0.05) and enhanced TDC (P < 0.05) with respect to the control group, indicating the impairment effect of AlCl3 on the PAL test. Moreover, two or five injections of exosomes significantly increased STL and decreased TDC compared with the AlCl3 group. These data suggest exosome injections could protect against the deterioration effect of AlCl3 on passive avoidance learning. Furthermore, two or five injections of exosomes into the saline-treated group had no significant effect on passive avoidance learning compared to the control group (P > 0.05) (Fig 6-7).

### Effects of treatments on hippocampal APP gene expression

**Fig 8:**
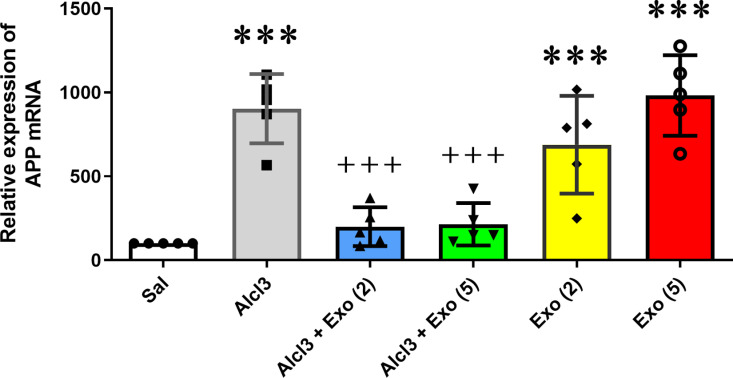
Effect of different treatments on relative APP gene expression in the hippocampus. APP mRNA levels were amplified by Alcl3 treatment compared to the control group (p < 0.001). Two or five injections of exosomes protected against ALCL3-induced enhancement of APP gene expression (p < 001). However, exosome injections in healthy rats increased hippocampal relative APP gene expression compared to the saline group (p < 0.001). Each value represents mean ± SEM, n = 5, *** p < 0.001 vs. saline group ^+++^p < 0.001 vs. Alc3 group. Sal, Saline; Exo [2], Two injections of exosomes; Exo [5], Five injections of exosomes.

Hippocampal APP mRNA was evaluated by RT-PCR for all six experimental groups (Fig 8). One-way ANOVA indicated a significant difference in the APP mRNA expression between the different groups (F (5,24) =26.51, p < 0.0001). Furthermore, AlCl3 treatment significantly amplified relative hippocampal APP gene expression compared to the control group (p < 0.001). Two or five injections of exosomes significantly decreased APP gene expression with respect to the Alcl3 group (p < 0.001). These results suggest the protective effect of MSC-extracted exosomes on deteriorations induced by AlCl3 on APP gene expression. Considering the role of the APP gene in Alzheimer’s disease, possibly MSC-extracted exosomes by reducing the expression of this gene protect against AlCl3-induced Alzheimer’s disease. However, our results showed that two (p < 0.05) or five (p < 0.01) injections of exosomes to saline-treated rats significantly increased hippocampal APP gene expression with respect to the control group.

### Histological results

**Fig 9:**
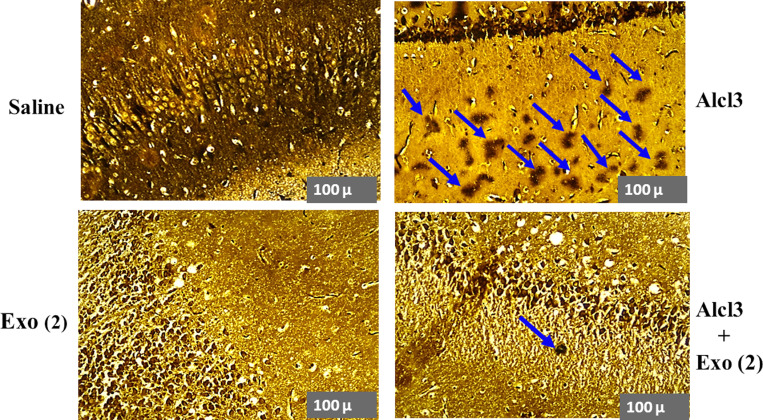
Silver-stained hippocampus sections of experimental groups. The blue arrows indicated the Aβ plaques. Induction of Alzheimer’s disease using Alcl3 severely stimulated Aβ Plaques formation. Two administrations of exosomes in Alcl3-treated rats obviously prevented Aβ plaques deposition. Bar = 100µm. Exo [2], Two injections of exosomes.

Hippocampal tissues were examined after silver staining to evaluate Aβ plaque deposition. AlCl3-treated animals obviously showed the formation of β-amyloid plaques in the hippocampus compared with the hippocampus of saline-treated rats (normal animals). The hippocampus from AlCl3 animals that received two injections of exosomes (AlCl3 – exo [2] group), revealed a protective effect of MSC-extracted exosome against Aβ plaque deposition. Also, in the saline-exosome treated sample, we could not find Aβ plaques (Fig 9).

## Discussion

Different studies have established the effect of heavy metals on neurodegenerative diseases such as AD. Al is one of the main heavy metals and humans are massively exposed to it. This element is present in the air, dust, water, food, medicines, flavored drinks, food additives, cosmetics, cooking dishes, etc [37]. Moreover, it has been reported that Al binds to some amino acids of APP and stimulates the organization of Aβ sheets [38]. Accumulation of insoluble beta-amyloid protein can lead to Alzheimer’s disease [39]. It seems, there is a relation between Aluminum and the risk of AD and other neurodegenerative diseases [40]. Moreover, using Alcl3 is a common method for the induction of Alzheimer’s disease animal models [15]. Ming-Hong Weng et al. (2020) have shown that the Alcl3 deteriorated memory capacity and increased expression of AD-related proteins in Alcl3-treated rats [41]. Amanzadeh et al. (2021) also have shown that injection of AlCl3 caused cognitive impairment and reduction of spatial memory in the Morris water maze test. They showed that rats treated with AlCl3 performed weaker than the control group during the stay in the target quarter in MWM [42]. In the present study, for induction of AD, rats received AlCl3 with a dose of 100 mg/kg for sixty days [42,43]. Our data were in line with previous results and showed that chronic AlCl3 treatment caused spatial memory and passive avoidance learning impairment, suggesting AD-like symptoms in rats. Spatial memory was evaluated by Morris Water Maze and according to the results, AlCl3 significantly decreased staying time in the target quarter. Also, our data indicated a significant impairment effect of AlCl3 on PAL retention.

Furthermore, atrophy and shrinkage of the brain are the symptoms of AD. Ali A. Azza et al. (2016) have shown that Al intake at doses of 70 and 100 mg/kg is associated with the destruction of hippocampal neurons and brain atrophy [44]. Our data are in line with previous studies and showed that the rate of brain weight to body weight declined in the animals that received AlCl3 (100mg/kg) for 60 days which indicates brain atrophy.

Cleavage of amyloid precursor protein (APP) causes the formation of Amyloid-β (Aβ) peptides. Aggregation of Aβ peptides especially Aβ42 can lead to the formation of Aβ plaques and AD disease [11]. It has been indicated that excess APP gene expression may amplify the risk of AD [45]. Animal studies have indicated a relation between AlCl3, enhancement of APP gene expression, and induction of AD-like disease [42,46]. Furthermore, studies by Mashoque et al. (2018) have shown that Al causes abnormal expression of the APP gene in laboratory samples, and abnormal expression of the APP can lead to the production of more amyloid beta (Aβ) peptide and then increase its accumulation [10]. Our molecular studies confirmed previous studies and revealed upregulation of APP gene expression in the hippocampus of AlCl3-treated rats.

β-Amyloid plaques (Aβ) are among the well-known hallmarks of AD, and the hippocampus is one of the first damaged regions in AD disease [47]. In this study, Aβ plaques were evaluated in the hippocampus using silver staining as one accepted tool in neuropathological investigations [35,48]. As expected, receiving AlCl3 (100 mg/kg) for two months caused the formation of Aβ plaques in the hippocampus of the animal model of AD.

Mesenchymal stem cell therapy has been noticed in neurological diseases for over a few decades [49]. For example, it has been reported that MSC transplantation could decline spatial memory impairments, Aβ plaque formation, and neuronal cell death in animal AD models [50,51]. There is growing evidence that indicates the effect of stem cells mostly mediates by secretion of exosomes or soluble paracrine agents [49,52]. Using exosomes in the treatment of diseases has several advantages over cell therapy including lower immunogenicity, more safety, the impossibility of direct tumor formation, and easy migration to the target organ without trapping in the lung microvascular [52–54]. Furthermore, exosomes are nano-v vesicles that can easily cross the brain barrier and deliver different therapeutic agents to the nervous system [21].

Accumulating studies have revealed that exosomes have a key role in different cell-to-cell pathways and can affect their interaction under various conditions [49]. Growing studies have shown the neuroprotective effects of MSC-derived exosomes. Zhang et al. (2014) have shown that intravascular injection of exosome-derived from MSC can increase axonal density and help to improve nerve regeneration in the cerebral cortex [55]. Ueno et al (2020) showed that exosomes can be potential therapeutic candidates for improving functional recovery in stroke survivors [56]. Also, one report in 2020 revealed the neuroprotective effect of intranasal injection of MSC-derived extracellular vesicles [57]. However, the current knowledge about the role of MSCs and exosomes secreted from them in the treatment of diseases is insufficient. Some studies have shown dual properties for them including, promoting or inhibiting effects on some diseases. The dual role of mesenchymal stem cells in the development or inhibition of apoptosis or cancer is mainly related to various factors such as protein, miRNA, lncRNA, and cytokines encapsulated in their derived exosomes [58]. Moreover, Rosenberger et al. (2019) have shown that exosomes can cause death and cytotoxicity or induce apoptosis in endothelial cells [59].

In this study, intravenous injection of MSC-extracted exosomes (150µg/µl) was performed. In two groups of rats that were under treatment AlCl3, they received exosomes between days 21 and 33. Our results showed that exosomes blocked the impairment effect of AlCl3 on the rate of brain weight to body weight. This result confirmed the protective effect of exosomes in preventing brain atrophy and neutralizing the destructive effects of AlCl3. The results of behavioral tests also confirmed the preventive effect of MSC-exosomes against the destructive effects of AlCl3. In the passive avoidance test, receiving exosomes significantly decreased the STL and increased the TDC compared to the AlCl3 group. However, in the MVM test, two injections of exosomes were more protective and significantly increased the time spent in the target quadrant to the AlCl3 group. Our results implied that there was no significant between two or five injections of exosomes on different evaluated parameters except spatial memory that injections with a limited number were more effective. Although there are studies that have reported positive effects of 8 injections of normoxic-MSC extracted exosomes (150µg/µl) on special memory in APP/PS1 transgenic mice. These contradictory results can be due to differences in the methods of experiments [28]. Anyway, it seems the number of exosomes injected to achieve the best response is important and needs more studies.

To reveal how exosomes affect the mechanism of memory, we evaluated the effect of exosomes on APP gene expression. Our molecular data revealed that exosomes significantly decreased the stimulating effect of AlCl3 on APP gene expression. It seems at least part of the MSC-exosomes mechanism in neutralizing the effect of AlCl3 in AD progress is mediated by its effect on APP gene expression. In vitro studies have shown that exosomes extracted from the AD mouse brain stimulate APP gene expression. It seems exosomal miRNAs have a critical role in this subject. Recently, it has been indicated that exosomes extracted from healthy neurons contain a high amount of APP-inhibiting agents such as miR-185-5p [60].

Our histological results confirmed molecular data and showed that exosome injection could obviously decrease and almost block AlCl3-induced β-Amyloid plaque formation. As mentioned in previous parts, different studies have evaluated the effect of exosomes on different neurodegenerative diseases. However, there is no report on the effect of exosomes in healthy subjects. Moreover, in this study, we also examined the effect of MSC-extracted exosomes on saline-treated rats. Contrary to what was expected, exosome injection impaired spatial memory and increased APP gene expression in healthy rats compared with the control group. However, Aβ plaques were not revealed in saline-exosome-treated rats. It seems, that in healthy conditions exosome injection caused some imbalanced conditions which led to negative effects such as disruption in spatial memory and augments of APP gene expression. It is suggested that the upregulation of APP mRNA was not at the level that caused Aβ peptides’ obvious aggregation.

Exosomes carry various miRNAs. Most miRNAs can target and regulate the expression of several genes simultaneously that play an important role in many biological processes and contribute to specific pathways related to cell death and cell growth, and pathways related to fibrosis such as Wnt signaling, PDGF, TGF-β, and cellular communication as well as affect the progression of various diseases [59,61]. According to our results, it seems that in damaged cells factors released from cells and exosome compounds can create active complexes for the regeneration and proliferation of peripheral stem cells, while in healthy cells, control factors prevent the effect of exosomes. Also, exosome miRNAs may inhibit the normal activities of the healthy cell by altering different mRNA expressions that need more studies. Moreover, regarding to our results, exosome injection cannot be recommended as a Memory enhancer in healthy conditions.

## Conclusion

We revealed that BM-MSC extracted exosomes prevented AlCl3-induced enhancement of hippocampal APP gene expression and beta-amyloid plaque formation and impairment of PAL and spatial memory and there was no difference between two- or five-times injections of exosomes. It seems these exosomes could be a candidate to prevent AD symptoms. However, more studies are needed before clinical usage. Also, according to our results using these exosomes is not recommended in the healthy subjects as a memory enhancer.

## Supporting information

S1 Data Dataset. (XLSX)

S1 Image S1_raw image. (PDF)
