# Supplementary figures and images for: Protective potential of BM-MSC extracted Exosomes in a rat model of Alzheimer’s disease
Source: PLoS One. 2025 May 6;20(5):e0320883. doi: 10.1371/journal.pone.0320883 (PMC12054907; doi:10.1371/journal.pone.0320883)

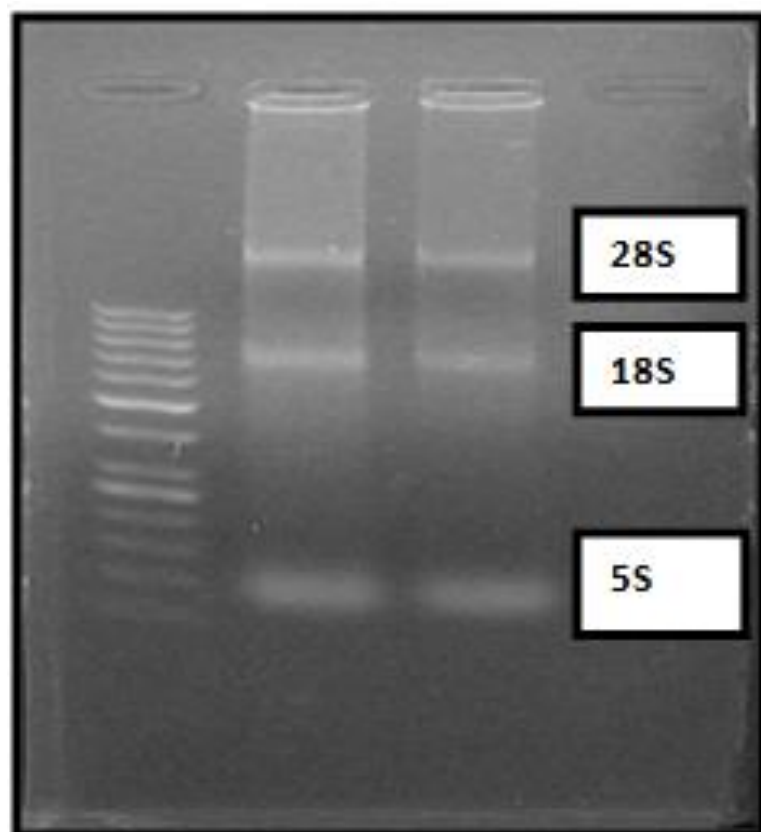

Supplement: S1 Image — (PDF) [file pone.0320883.s002.pdf]
